# Monoclonal Antibodies against Epidermal Growth Factor Receptor in Solid Tumors

**DOI:** 10.1155/2012/291421

**Published:** 2012-12-24

**Authors:** Nabil F. Saba, Sue S. Yom, Missak Haigentz, Bassel El-Rayes

**Affiliations:** ^1^Department of Hematology and Medical Oncology, Emory University, Atlanta, GA 30322, USA; ^2^Department of Radiation Oncology, University of California, San Francisco, CA 94143, USA; ^3^Department of Medicine, Montefiore Medical Center, Albert Einstein College of Medicine, Bronx, NY 10461, USA

The discovery of tumor-specific molecular alterations has ushered in a new era of oncology, leading to the development of novel therapeutic agents specific to cancer subtypes. Since the discovery of the Philadelphia chromosome in 1960, focused efforts to inhibit and target abnormal cell signal transduction have transformed the treatment and outcome for numerous cancer types, increasingly including solid tumors.

Targeting of the epidermal growth factor receptor (EGFR) has been at the forefront of efforts to develop compounds modulating signaling pathways and altering clinical outcomes for patients with various types of solid tumors. Extensive research efforts focusing on the EGFR pathway in different solid tumor types have resulted in recent approval of a number of novel therapeutic agents for locally advanced as well as metastatic disease. In this issue, we offer an overview of established and evolving EGFR monoclonal antibody (MoAb) applications in common solid tumors, with a dual focus on approved and novel agents in clinical development.

In squamous cell carcinoma of the head and neck, EGFR MoAbs are now an integral component of the standard of care, either in combination with radiotherapy for primary disease or with platinum-based chemotherapy for recurrent or metastatic disease. These breakthrough developments, which have occurred only over the past several years, arguably represent the first major advance in systemic therapy for head and neck cancer in the past 30 years. Novel and potentially more effective agents with less side effects are in development.

In colorectal cancer, the use of EGFR MoAbs has become an integral component in the treatment of advanced disease, but careful selection of patients based on KRAS mutation status is now a component of practice guidelines. Similarly, inhibition of Her2 has been an approved standard in the treatment of Her-2 overexpressing or amplified breast cancer. The documented resistance to trastuzumab has led the way in developing novel monoclonal antibodies and other Her2 targeted agents for this disease.

Although EGFR MoAbs have been under clinical investigation in lung cancer, the frequently observed somatic kinase domain activating mutations affecting the EGFR oncogene has led to widespread clinical use of EGFR tyrosine kinase inhibitors (TKIs). Recently, de novo resistance mechanisms have been described. Given the very active research in this area and its broader implication on EGFR inhibition overall, we have included a comprehensive and timely review of this topic. Finally, other Erb B family receptors such as Her3 are now emerging as important modulators of resistance to EGFR inhibitors, and several Her3 targeted agents are now in clinical development.

In addition to presenting an overview of EGFR MoAb applications, we have also reviewed issues pertinent to toxicities, mechanisms of resistance, and interactions with other pathways. We believe that the evolving knowledge and therapeutic focus on the EGFR and other pathways will lead to the development of novel targeted agents with increased capacity to reverse the oncogenic process of solid tumors.

*Nabil F. Saba* *Sue S. Yom* *Missak Haigentz* *Bassel El-Rayes*

